# Environmental life cycle assessment of production of the high intensity sweetener steviol glycosides from *Stevia rebaudiana* leaf grown in Europe: The SWEET project

**DOI:** 10.1007/s11367-022-02127-9

**Published:** 2023-01-14

**Authors:** J. Suckling, S. Morse, R. Murphy, S. Astley, J. C. G. Halford, J. A. Harrold, A. Le-Bail, E. Koukouna, H. Musinovic, J. Perret, A. Raben, M. Roe, J. Scholten, C. Scott, C. Stamatis, C. Westbroek

**Affiliations:** 1 Centre for Environment and Sustainability, University of Surrey, Guildford, UK; 2 EuroFIR AISBL, Brussels, Belgium; 3 School of Psychology, University of Leeds, Leeds, UK; 4 Department of Psychology, University of Liverpool, Liverpool, UK; 5 ONIRIS, UMR GEPEA CNRS 6144, Nantes, France; 6 Blonk Consultants, Gouda, The Netherlands; 7 Stevia Natura, Auvergne, France; 8 Department of Nutrition, Exercise and Sports, University of Copenhagen, Copenhagen, Denmark; 9 Clinical Research, Copenhagen University Hospital – Steno Diabetes Center Copenhagen, Herlev, Denmark; 10 Cargill, Plymouth, MN USA; 11 Stevia Hellas, Lamia, Greece

**Keywords:** Life cycle assessment, High intensity sweeteners, Stevia, Steviol glycosides, *Stevia rebaudiana*

## Abstract

**Purpose:**

There is an increasing interest in the use of non-nutritive sweeteners to replace added sugar in food and beverage products for reasons of improving consumer health. Much work has been done to understand safety of sweeteners, but very little on sustainability. To address that gap, this study presents the results of a life cycle assessment (LCA) of production of rebaudioside A 60%, 95% pure (RA60) steviol glycoside mix from *Stevia rebaudiana* leaf grown in Europe.

**Methods:**

An attributional cradle-to-factory-gate life cycle assessment was conducted on growing of stevia leaves and extraction of steviol glycosides in Europe. Primary data were used from a case study supply chain. Results are reported in impact categories from the ReCiPe 2016 (H) method, with focus given to global warming potential, freshwater eutrophication, water consumption, and land use. Impacts are expressed both in terms of production mass and sweetness equivalence, a common metric for understanding high intensity sweetener potency. Sweetness equivalence of RA60 is typically 200 to 300 times that of sugar. Comparison of environmental impact is made to sugar (sucrose) produced from both cane and beets. The research is part of the EU project SWEET (sweeteners and sweetness enhancers: impact on health, obesity, safety, and sustainability).

**Results and discussion:**

Global warming potential for production of RA60 was found to be 20.25 kgCO_2_-eq/kg_RA60_ on a mass basis and 0.081 kgCO_2_-eq/kg_SE_ on a sweetness equivalence basis. Field production of stevia leaves was found to be the main source of impact for most impact categories, and for all four focus categories. Extraction of the RA60 was the main source of impact for the others. Leaf processing and seedling propagation were minor contributors to life cycle impact. Removal of international transport from the supply chain reduced global warming potential by 18.8%. Compared with sugar on a sweetness equivalence basis, RA60 has approximately 5.7% to 10.2% the impact for global warming potential, 5.6% to 7.2% the impact for land use, and is lower across most other impact categories.

**Conclusion:**

This is the first LCA of steviol glycoside mix RA60 produced from leaf in Europe. The results indicate that RA60 can be used to reduce environmental impact of providing a sweet taste by replacing sugar across all impact categories. However, it is important to note that specific formulations in which RA60 is used will have a bearing on the final environmental impact of any food or beverage products. For solid foods, this requires further research.

**Supplementary Information:**

The online version contains supplementary material available at 10.1007/s11367-022-02127-9.

## Introduction

There is increasing concern regarding the role of added sugar in non-communicable diseases such as diabetes and obesity (Johnson et al. 2017) or tooth decay (Vaghela et al. 2020). As such there is also growing interest in the potential to use non-nutritive sweeteners (NNS) as replacements for added sugar and the potential for health benefits as associated with weight loss (O'Connor et al. 2021; McGlynn et al. 2022; Rios-Leyvraz and Montez 2022). To be used in food or beverages products within the European Union, food additives including NNS must undergo a rigorous approval procedure as outlined in Regulation EC 1333/2008. To be accepted, research must show that NNS are safe to consume at the levels proposed for use (Younes et al. 2020).

One such NNS and the focus for this study are steviol glycosides (SGs), which are extracted from the leaves of *Stevia rebaudiana* (Ciriminna et al. 2019), hereafter “stevia.” The plant has been known in Paraguay for its sweet tasting leaves for thousands of years (Angelini and Tavarini 2014) and is now grown around the world, with the majority of production in China (Stamatis and Perret 2015). There are numerous SGs present in varying quantities in the leaves of the stevia (Ciriminna et al. 2019). The most abundant is stevioside, followed by rebaudioside A (Reb A), and many other rebaudiosides named with letter identifiers, for example, Reb C, D, and M (Ciriminna et al. 2019). The different glycoside molecules have the same steviol backbone, onto which varying numbers of glucose molecules are attached (Oehme et al. 2017). The resulting molecules each have a much greater sweetness per unit mass than sugar. The sweetness of each Reb variant is different but tend to be in the region of 200–300 times that of sugar, depending on application in food or beverage (Cardello et al. 1999; Wallin 2004; Prakash et al. 2014). This means that 1 kg of sugar can be replaced by a much smaller mass of SGs. Sucrose, hereafter ‘sugar,’ has a sweetness equivalence (SE) of 1. Therefore, both approximately 4 g SGs and 1 kg sugar provide 1 kg_SE_.

In the EU, SGs are classified as a food additive E960 and allowed as an ingredient if certain criteria are met (European Union 2012; Younes et al. 2019). One such criteria is that the maximum acceptable daily intake (ADI) is 4 mg/kg body weight. One of the SG mixtures currently permitted within the EU contains at least 60% Reb A at a purity of 95% (RA60), which means that 5% of the total mix is impurities from the plants and extraction process and the remaining 35% is other rebaudiosides.

Studies required for SGs to be considered safe focus on toxicity and numerous examples are listed by Younes et al. (2020). Similarly, studies relating to the potential for SGs to combat non-communicable diseases are also numerous but more diverse in their outcomes (Ahmad et al. 2020; Rios-Leyvraz and Montez 2022). However, to date, there has been little research into the environmental impact of *any* NNS, let alone SGs. The authors are aware of four studies relating to SGs: an LCA for Reb M and Reb D from sugar by fermentation (Cargill 2021); a carbon and water footprint of stevia sweeteners (PureCircle 2015); an LCA of pure Reb A and Reb M from leaf and fermentation methods (Milovanoff and Kicak 2022); and an LCA for steviol glycoside powder from leaf (Gantelas et al. 2022). This study builds upon those works by providing an LCA of 60% stevia rebaudioside A, 95% purity steviol glycoside mix (RA60) from leaves of *S. rebaudiana*.

This paper is organized as follows. An overview of the production process and the LCA methodology used is provided in Section 2. Detailed information and inventory data relating to the life cycle assessment are presented in Section 3. Results for production of 1 kg SGs are given in Section 4, and sensitivity analysis in Section 5. Discussion is provided in Section 6 and includes a comparison of environmental impact of SGs to sugar on a sweetness equivalence basis. Finally, concluding remarks are made in Section 7.

## Overview of the production process and LCA

The focus for this LCA was production of RA60 from stevia leaves grown in central Greece and extracted in France. For the purposes of this LCA, production process was divided into four phases: seedling propagation, field cultivation, leaf processing, and extraction.

The stevia is grown by a cooperative of farmers who have diversified and converted agricultural land from growing tobacco to growing stevia, which is viewed as a viable alternative crop (Kienle et al. 2015). In total, 60 farmers cultivate 50 hectares of land, an average of 0.83 ha per farmer. They use a mixture of farming techniques, including manual and mechanical methods, which differ among the farmers. However, many of the techniques and methods are the same for growing stevia as they were for growing tobacco (interview data).

The cooperative was established in 2012 and has produced RA60 in collaboration with a company in France since 2016. A total of approximately 180 t dried stevia leaves are produced annually, from which 40% is sold for RA60 extraction. The other 60% is sold to companies who sell stevia-based teas and infusions. The production of both products is the same up to the point where the dried leaves are cleaned. The leaves destined for RA60 extraction do not undergo any further processing, whereas those destined for tea/infusions go through further sorting for cut size and milling. Therefore, the mass of stevia produced does not influence allocation of the impacts as they are the same for both products up to the point of separation.

Stevia seedlings are propagated in a greenhouse for 60 days until they are ready for transplanting into fields. During those 60 days, they are trimmed twice with a lawnmower to encourage leaf growth. The seedlings are planted in field at a density of 60,000 to 70,000 plants/ha. The first harvest can take place after 60–90 days, and the plants are grown for 5 years and harvested once a year, after which time they are replaced. The yield of dried leaves in the first year is approximately 2 t/ha, and 4 t/ha for years two to five, giving an average over the 5 years of 3.6 t/ha.

The leaves are dried on the farms before being transported to a central processing facility. At this facility, they are cleaned to remove remaining stalks and other debris. Finally, they are transported to the extraction factory in France where the RA60 is produced and then sold to food and drink manufacturers.

### Goal and scope

The goal of the LCA was to assess the environmental impact of producing 1 kg Reb A 60%, 95% purity (E960) steviol glycoside mix from stevia grown in Europe. The leaves are sourced from and processed in Greece, before the RA60 is extracted in France.

The study included all foreground activities associated with stevia growth, processing of leaves to remove debris, extracting the RA60 and transport in between locations. Background activities were also included for inputs such as energy, transport, infrastructure, and other materials. Disposal of wastes was also included.

The LCA was undertaken in line with ISO 14040:2006 (ISO 2010a) and ISO 14044:2006 (ISO 2010b) guidelines.

### System boundaries

**Fig. 1:**
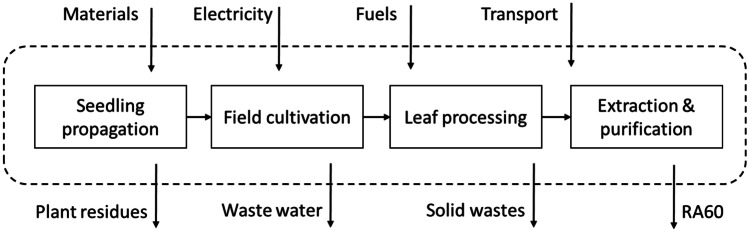
System boundaries for the life cycle assessment

The LCA system boundary is shown in Fig. 1 and includes phases of the following: seedling propagation in a greenhouse; planting, growing, harvesting and drying of the stevia leaves (field cultivation); processing of the dried leaves into a form suitable for RA60 extraction (leaf processing); and extraction and purification of the RA60 from the leaves. All upstream inputs, such as electricity, other energy inputs, and machinery and infrastructure, were included. Outputs such as by-products and wastes were also included.

The assessment was conducted using SimaPro 9.3 software and ecoinvent 3.8 and Agri-footprint 6.1 databases. The primary characterization method used was ReCiPe 2016 Midpoint (Hierarchist) (Huijbregts et al. 2016). Environmental impact within all the impact categories of the ReCiPe 2016 method is reported, with focus given to global warming potential, water consumption, land use, and freshwater eutrophication due to their relevance to production of plant-based food ingredients.

Primary data were collected through interview with producers of both the stevia and the RA60 glycoside mix. Site visits were not possible over the study period due to COVID-19 public health restrictions. Therefore, all interviews were conducted remotely, and recorded, and further familiarization of the locales and processes conducted through literature and internet searches. Uncertainty was modelled where data were available.

### Functional unit

The LCA study was cradle-to-gate, and the functional unit “production of 1 kg of RA60 SGs, at a 95% purity.” The “gate” in this case was the exit from the RA60 production factory. Transport of the RA60 to customers and use of RA60 in food and beverage formulations was omitted as being out of scope. The use of SGs in formulations is dependent upon other factors that are discussed later in this manuscript.

Mass was used as the functional unit because of its international standard, but it does not represent the functional use of RA60, or indeed any other NNS. Such substances are perceived as being much sweeter than sugar and, therefore, are often described in terms of “sucrose equivalence” (SE). In short, 0.004 kg RA60 is approximately equivalent to 1 kg sugar in terms of sweetness perception. Therefore, the results are also presented in terms of 1 kg_SE_ RA60 (0.004 kg RA60). However, it should be noted that these results are used as a guide, because RA60 sweetness equivalence varies depending on use and concentration, and is typically cited as being between 200 and 300 (Cardello et al. 1999; Wallin 2004). The effect of a varying sweetness equivalence is also presented.

### Allocation

Allocation of impact was on an economic basis. The only product of value within the life cycle is stevia leaves and subsequently RA60. All other plant residues and processing outputs have no value and are returned to the fields or treated in wastewater treatment plants. The only point in the life cycle when co-products are produced is when processed leaves are separated for either RA60 extraction or tea/infusion products. However, at the point of separation, their economic value is the same, and further value is only added by additional processing of the two streams. Therefore, mass and economic value are equivalent at this point in the life cycle.

The cut-off method is used to deal with recycling of materials. Using this method, all materials for recycling leave the system without any environmental burden. Likewise, recycled materials entering the system do not carry in any environmental burden, other than those associated with recycling.

## Life cycle inventory data

The case study cooperative sources stevia from farmers who operate on multiple scales and use different production processes. Therefore, a representative life cycle of a “typical” production method was used. Moreover, much of the infrastructure and equipment used for the stevia cultivation is the same as that previously used for tobacco cultivation. For simplicity, it was assumed that the infrastructure and machinery have only been used for stevia cultivation, and impact allocated solely to stevia production. In addition, the land used was diversified from tobacco to stevia but remains agricultural for the purposes of land use. Therefore, the net assumption was that stevia cultivation on this land has reached the steady state of an established business. Inventory data are given for each life cycle phase in Supplementary Material, Tables S5–S9.

### Phase: seedling propagation

Seedlings are propagated in a greenhouse, which is made from a steel frame supporting a plastic film. No artificial lighting or temperature control is used. Seedlings are grown from seed for 60 days before being planted in the field. Transport from greenhouse to the field was accounted for as part of the field cultivation process. The greenhouse has a lifetime of 20 years and, therefore, is used for five cycles of stevia propagation (it was assumed that it is not used for other purposes during that time).

The seeds are planted into expanded polystyrene trays which have 247 holes and a size of 60 cm by 40 cm by 7 cm. Each tray is filled with 0.005 m^3^ compost and seeds sowed using a mechanical device that consumes 4.5 kWh/ha of seedlings sown. Seedling density in the trays is equivalent to 10.3 million plants per hectare and the greenhouse, in this instance, has a capacity for 560 boxes.

The seedlings are topped twice with a lawnmower mounted on a gantry during propagation to promote leaf growth. Seedling topping is represented by a proxy process of a rotary lawnmower to a total area of 0.48 m^2^ per 247 seedlings.

The polystyrene trays are floated on a waterbed and tightly packed to minimize exposed water surface and hence minimize evaporation. Approximately 2.5 L of water are used per tray per 60-day period. The water is held within a bed that is lined with plastic pond-liner. The liner is 1-mm thick and covers an area of 168 m^2^ for 560 trays. It is assumed used 5 times.

Fertilizer is used in the water at a rate of 110 mg N/liter. However, the type of fertilizer varies between farmers, so a generic NPK 15–15-15 fertilizer was assumed. No pesticides were used in this phase.

### Phase: field cultivation

The seedlings are transplanted to the field using a semi-mechanized process. A tractor carries a tobacco planting machine on which four people sit to feed seedlings from the trays into four planting mechanisms. Seedlings are planted at a density of 65,000 plants/ha with a range of 60,000–70,000 plants/ha, and a planting rate of 16 h/ha. The polystyrene seedling trays are recycled.

The fields are watered through drip irrigation, and 150–200 m^3^ water per tonne of dried leaf material is used. PVC irrigation pipes are used with an approximate length of 14.3 km/ha and lifetime of 10 years. Fertilizer is applied at 200 kg/ha twice during the first year, and once per year thereafter. It is spread at a rate of 1 h/ha. As before, a generic NPK 15–15-15 fertilizer was assumed, as the fertilizer used will vary depending on the soil type at any given field location. Fungicide in the form of copper sulphate solution is applied at 4.219 kg/ha (1.5 kg_MCE_/ha, mole copper equivalent), twice per year, at a rate of 1 h/ha. An uptake of 10 ppm (per annum) is estimated for the plants, and the rest of the copper sulfate remains in the soil.

The fields are weeded mechanically three times a year using a tractor drawn mechanism. Weeding takes place at a rate of 3 h/ha.

Stevia leaves are harvested once a year, using a tractor drawn harvester at a rate of 16 h/ha. In the first year, the yield of dried leaves is approximately 2 t/ha and then 4 t/ha for years 2 to 5. This leads to an average yield of 3.6 t/ha/yr. Any crop residues are spread back to land. Emissions of greenhouse gases and heavy metals from fertilizer use and crop residues are modelled as per the Agri-footprint 6 methodology (Blonk et al. 2022). Data relating to NPK content of the stevia leaves and stems were taken from Angelini and Tavarini (2014). Data relating to heavy metal uptake were not available for stevia; therefore, data for chicory leaves from Table 3.10 in Blonk et al. (2022) were used as a proxy. Emissions data are given in Supplementary Material, Table S5.

For each of planting, weeding, harvesting, and spreading operations, a tractor is used to pull appropriate equipment. The tractor was assumed to have a 40 kW power plant; however, it is not known at what intensity the power plant operates. Therefore, an energy consumption of 4076.07 kWh/5-years was estimated for the tractor, with a range from 1872.15 kWh/5-years (Kolator 2021) up to 6280 kWh/5-years (40 kW). The tractors and all equipment were assumed to have a lifetime of 25 years.

After harvest, leaves are put into a dryer that consumes 300 kWh/t dried leaves. The source of the energy is electricity from the Greek national grid. The drying cabinet is the same as that used for drying tobacco leaves: a shed with a lifetime of 25 years. There is potential for the stevia to be dried without using energy, and this was explored in the sensitivity analysis. Dried stevia leaves are put into reusable jute bags for transporting to the processing factory. Any additional plant offcuts from this stage are ploughed back into the field.

The leaves are shipped to the processing facility that is located an average of 10 km from the fields. Different modes of transport are used and were cited in interview as being either by tractor and trailer, or personal vehicle, depending on the size of the farmer’s plot of land. To enable transport to be reported in terms of t/km, light commercial vehicles were used as a proxy to personal vehicles, which are usually reported in terms of “per km.” Therefore, a typical transport distance was estimated to be 0.01 t.km/kg dried leaves with a 50:50 split between the two modes of transport.

It should be noted that no weed control matting, or insecticides, are used in the fields.

### Phase: leaf processing

Dried stevia leaves are processed in a dedicated facility. First, leaves are cleaned in a zig-zag cleaner which separates them from stems and other falling material (such as stones or soil). The cleaner has a capacity of 150 kg/hour and consumes 0.08 kWh/kg. Approximately 20 to 30 kg of materials are separated per 150 kg, of which about 80% are stems and 20% other materials. Removed materials are spread back to land and emissions calculated as for crop residues from the field cultivation phase, Section 3.2. Emissions data are given in Supplementary Material, Table S5.

All the processed leaves are packed into cardboard boxes for shipping. The capacity of the boxes is 20 kg, and the cardboard has a mass of approximately 793 g per box. After processing, leaves are transported to the factory for extraction. Transport is a total of 17 t.km/kg dried leaves by 35 t truck and 0.9 t.km/kg of dried leaves by ferry (carrying the truck). The cardboard boxes are recycled in the locale of the extraction factory.

### Phase: extraction

On average, 10 kg of dried stevia leaves are used to produce 1 kg of purified SGs (herein written as kg_RA60_). The total SG content of leaves is approximately 13%, but not all is extracted, as some is lost during purification of the crude SG mix, resulting in a 10% effective SG content. The factory is a dedicated facility with a production capacity of 25 t SGs per year if operated during office hours and 50 t/year if operated on a 24/7 basis. It has a lifetime of at least 30 years.

The leaves are steeped in boiling water (10 l/kg leaves) to extract rebaudioside molecules, producing approximately 1 kg of RA60, and 9 kg of residual plant matter, which is taken by local farmers and spread onto fields. Water from the process is sent to the municipal sewer and treated in a wastewater treatment plant.

The crude SG mix is clarified using 0.02 kg/kg_RA60_ of calcium hydroxide and 0.02 kg/kg_RA60_ citric acid. The resulting flocculant mud is considered part of the residual plant matter (i.e., within the 9 kg plant matter) and is also spread to land. Again, emissions from the waste leaf matter are calculated using data from Angelini and Tavarini (2014) and methodology from Blonk et al. (2022). Emissions data are given in Supplementary Material, Table S5.

Next, the crude steviol glycoside solution is passed through chromatography columns to remove impurities. These contain silica resins, 500 L of which are used to separate 50 kg_RA60_ and have a lifetime of 3 years. Therefore, given the current production of 25 t/year of the factory, columns need to be replaced at a rate of 0.0132 kg/kg_RA60_. At the end of their useful life, resins are disposed of via appropriate contract waste disposal.

SGs are further purified using ethanol to crystallize glycoside molecules. Ethanol is used at a rate of 3 to 4 l/kg_RA60_ before being sent back to the manufacturer for repurification. Approximately 1% of ethanol is lost per cycle (ca. 0.03 l/kg_RA60_). The ethanol is assumed to be produced from fermentation of sugar from cane.

In total, 0.5 kWh/kg_RA60_ electricity and 0.5 kWh/kg_RA60_ gas is consumed during RA60 extraction. The electricity is drawn from the French national grid.

The resulting product is packaged in 10 kg quantities in plastic bags and two bags per cardboard box for shipping to customers. As before, the cardboard box was estimated to have a mass of 793 g. Transport out of the extraction factory was not included within this LCA.

### Assumptions

The following assumptions were made for the LCA:

1. Biogenic carbon was not accounted for as per IPCC recommendations for annual crops (IPCC 2019). It is acknowledged that the stevia plants are grown over a 5-year period and, therefore, are not annual, but neither is the carbon locked into the plants for an extended period. Therefore, it was assumed that all carbon absorbed during growth was emitted back to the atmosphere on timescales much shorter than the 100-year timescale used in the impact assessment.
2. Life cycle data can be expressed over the 5-year cultivation cycle. Nuance within this period was ignored, e.g., the effect of yield in the first year on production resource needs.
3. Production processes vary among individual farmers depending on scale; smaller scale production favors polytunnel seedling propagation, and manual field cultivation, whereas larger scale production favors greenhouse seedling propagation and mechanized field cultivation. Therefore, greenhouse production and mechanized field cultivation were taken as indicative for all production scales.
4. Final mass of RA60 extracted from the leaves is 10% of the initial dried leaf mass. This should be considered indicative, as different varieties of *S. rebaudiana* have different quantities of SGs.
5. Similarly, the harvest was assumed to be 3.6 t/ha of dried leaves, but as before, different varieties of *S. rebaudiana* have different leaf yields.

## Results

The LCA results are presented as a function of 1 kg_RA60_ mass, and four impact categories highlighted for discussion of environmental impact: global warming potential, freshwater eutrophication, land use, and water consumption. Full results are given in Table S1 in the supplementary information, showing environmental impact for each of the ReCiPe 2016 (H) mid-point impact categories.

**Fig. 2:**
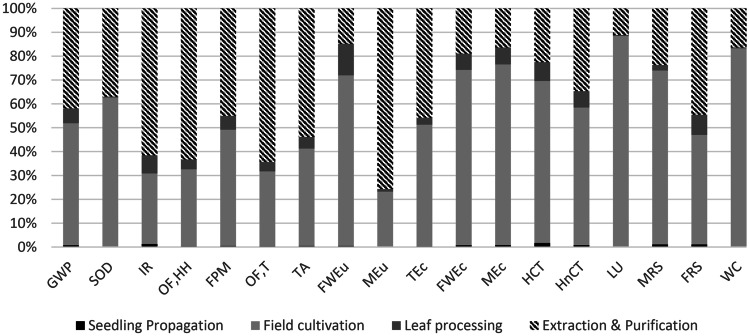
Relative impacts of each life cycle phase for all ReCiPe 2016 (H) impact categories. Phases shown are seedling propagation (black), field cultivation of leaves (light grey), leaf processing (dark grey), and SG extraction and purification (hashed). GWP, global warming potential; SOD, stratospheric ozone depletion; IR, ionizing radiation; OF,H, ozone formation, human health; FPM, fine particulate matter; OF,T, ozone formation, terrestrial; TA, terrestrial acidification; FWEu, freshwater eutrophication; TEc, terrestrial ecotoxicity; FWEc, freshwater ecotoxicity; MEc, marine ecotoxicity; HCT, human carcinogenic toxicity; HnCT, human non-carcinogenic toxicity; LU, lane use; MRS, mineral resource scarcity; FRS, fossil resource scarcity; WC, water consumption

**Fig. 3:**
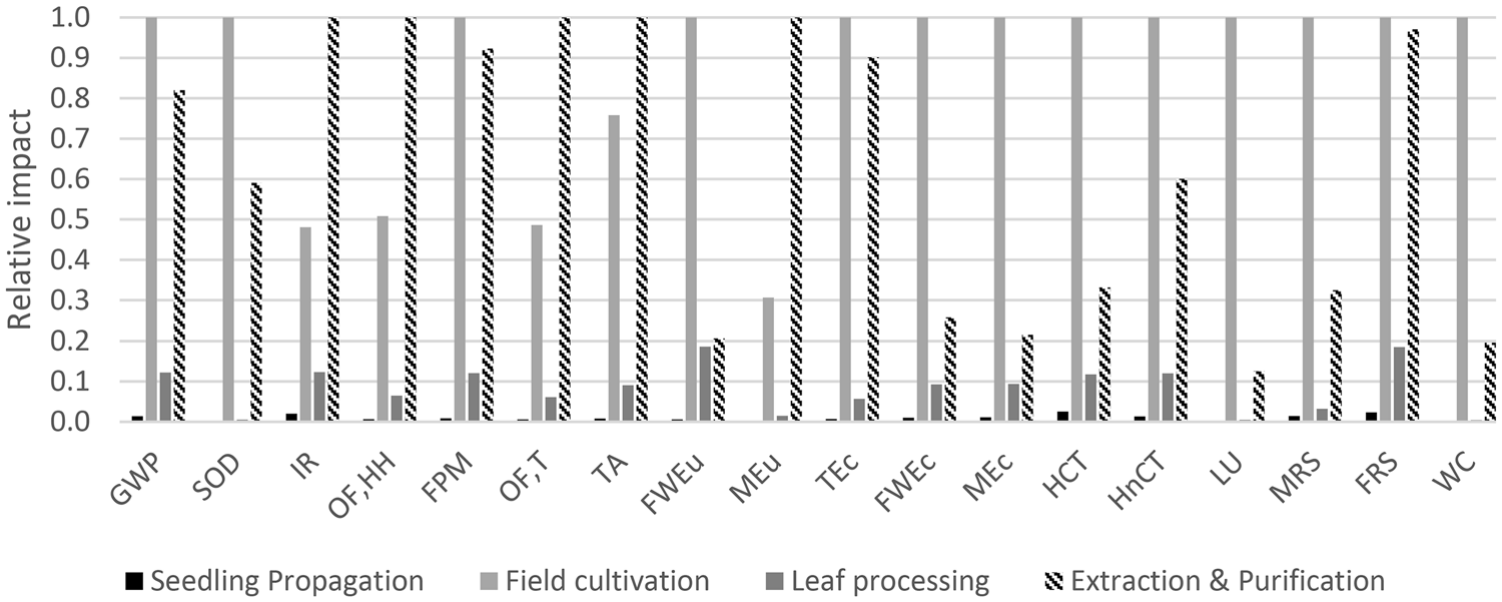
Impact of each life cycle phase normalized to the most impactful, for all ReCiPe 2016 (H) impact categories. Phases shown are seedling propagation (black), field production (light grey), leaf processing (dark grey), and RA60 extraction (hashed)

Figure 2 shows the relative impact of all life cycle phases for the four impact categories. The life cycle phases shown are seedling propagation (black), field cultivation (light grey), leaf processing (dark grey), and extraction and purification (hashed). Similarly, Fig. 3 shows the relative importance of each phase compared with the most impactful phase for each of the impact categories. Color coding is the same as per Fig. 2. Supporting data are given in Supplementary Information, Table S2.

Total global warming potential (GWP) is 20.25 kgCO_2_-eq/kg_RA60_ for the whole life cycle. Main sources of impact are field cultivation (10.35 kgCO_2_-eq/kg_RA60_) and extraction (8.49 kgCO_2_-eq/kg_RA60_). Within field production, main sources of impact are N emissions from fertilizer and crop residues (3.41 kgCO_2_-eq/kg_RA60_), the diesel used to power the tractor (1.20 kgCO_2_-eq/kg_RA60_) and electricity for drying the leaves (2.75 kgCO_2_-eq/kg_RA60_). Within extraction, main sources of impact are the freight lorry and ship transport from the processing facility to the extraction factory (3.75 kgCO_2_-eq/kg_RA60_), production of ethanol from fermentation used in purification (2.75 kgCO_2_-eq/kg_RA60_) and N emissions from waste leaf matter (1.29 kgCO_2_-eq/kg_RA60_).

Total water consumption (WC) is 2.53 m^3^/kg_RA60_ for the whole life cycle. Again, field production is the main source of environmental impact (2.11 m^3^/kg_RA60_), and almost all due to irrigation of stevia plants (2.06 m^3^/kg_RA60_). Within the extraction phase, once again, ethanol is the main source of impact (0.39 m^3^/kg_RA60_). It should also be noted that within the extraction phase 0.10 m^3^/kg_RA60_ of water is used, but 0.09 m^3^/kg_RA60_ of this is considered returned to the environment through wastewater treatment.

Total freshwater eutrophication (FWEu) is 1.44 × 10^−2^ kgP-eq/kg_RA60_ across the whole life cycle. The main source of impact is once again field production phase (1.03 × 10^−2^ kgP-eq/kg_RA60_), over half of which is derived from consumption of electricity (6.03 × 10^−3^ kgP-eq/kg_RA60_). The net impact of extraction is 2.13 × 10^−3^ kgP-eq/kg_RA60_, of which the majority comes from ethanol production (1.05 × 10^−3^ kgP-eq/kg_RA60_), and transport (6.85 × 10^−4^ kgP-eq/kg_RA60_). The processing accounts for 1.92 × 10^−3^ kgP-eq/kg_RA60_, of which most (1.61 × 10^−3^ kgP-eq/kg_RA60_) is due to consumption of electricity in cleaning machinery.

Total land use (LU) is 37.85 m^2^acrop-eq/kg_RA60_ across the whole life cycle. The majority of this is from field cultivation (33.49 m^2^acrop-eq/kg_RA60_). The next most impactful phase is extraction and purification at 4.19 m^2^acrop-eq/kg_RA60_ of which 3.98 m^2^acrop-eq/kg_RA60_ is due to production of ethanol. This skew in favor of field cultivation is due to the relatively low yield of stevia leaves per hectare per year (3.6 t/ha.yr).

Figure 3 shows relative impact of all impact categories across the four life cycle phases. Field cultivation is the main source of environmental impact for 12 impact categories, and RA60 extraction is the main source for the other six categories. Both processing and seedling propagation are relatively minor contributors to the total life cycle impact across all impact categories.

**Fig. 4:**
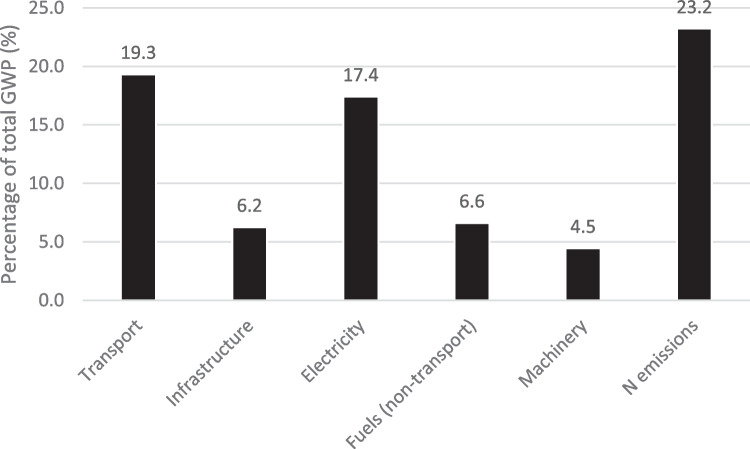
Impact attributable to different categories of foreground process

Finally, Fig. 4 shows the percentage of GWP attributable to different categories of foreground process. Impacts within background processes are not included in these numbers. The results indicate that N emissions from fertilizer application, crop residues, and waste leaf matter are the main contributors of GWP (23.2%). Next is transport (19.3%, mainly due to transport from processing facility to extraction factory), followed by electricity consumption (17.4%, mainly due to leaf drying). Impacts from fuels used in agricultural machinery account for 6.6%, and infrastructure (i.e., buildings or items such as irrigation pipes) accounts for 6.2%. Finally, machinery (i.e., tractors and other mechanical aids) accounts for 4.5%. These results indicate that electrification of transport and reduction of carbon intensity of the Greek electrical mix will both be significant contributors to reducing GWP of producing RA60.

### Scenario analysis

Changes were made to some parts of the life cycle inventory to explore their effect upon the results:

- Replacing electrically assisted forced drying with passive air drying. This is a process that can also affect the quality of SGs extracted (Lemus-Mondaca et al. 2018; Tellez et al. 2018).
- Moving location of RA60 extraction from France to Greece, to be closer to the cultivation location. Currently dried leaves are transported from Greece to France by road and sea freight, and transport is assumed to be an important contributor to GWP. Removing transport and changing location will help elucidate their contribution across all impact categories.
- Swapping ethanol derived from fermentation of sugar cane in extraction and purification process with ethanol derived from fossil sources, e.g., by hydrolysis of ethylene.

**Fig. 5:**
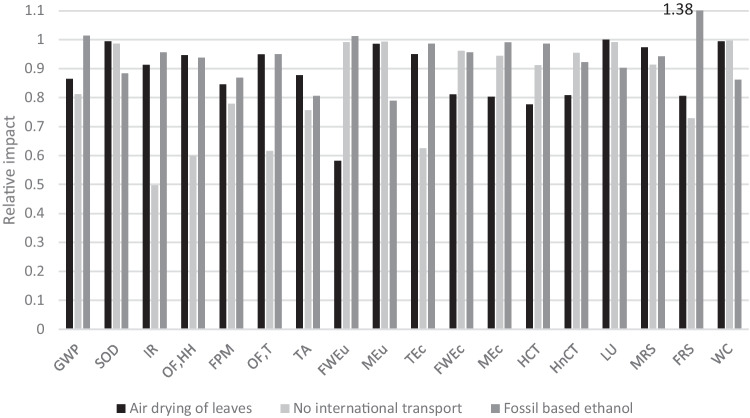
Relative impact change of altering parameters within the life cycle inventory; air drying of leaves (black), no international transport (light grey) and ethanol derived from fossil sources instead of fermentation (dark grey) are shown. FRS for fossil-based ethanol is greater than 1.1, at a value of 1.38 (marked on graph)

Results from the scenario analysis are shown in Fig. 5 as a relative change in impact compared with the default case used in the LCA study. The effects of air drying of leaves (black), removal of international transport (light grey), and use of fossil derived ethanol (dark grey) are shown. Supporting data are given in Supplementary Information, Table S3.

In the case of air drying of leaves, environmental impact is reduced across all impact categories. For instance, GWP is reduced by 13.6%, fossil resource scarcity (FRS) by 19.4%, and FWEu by 41.9% driven by the reduced demand for fossil fuels in electricity production in Greece. Conversely, LU reduces by less than 0.1%, mineral resource scarcity (MRS) by 2.7%, and WC by 0.6%, as these are dominated by impact from growing stevia, and not electricity consumption from drying the leaves.

Removal of international transport also reduces impact across all categories, e.g., GWP by 18.8% (reduction in transport distance), or ionizing radiation (IR) by 50.3% (due to replacement of French with Greek electricity mix for extraction of RA60). Again, some categories only have small changes, such as LU (0.8%), or WC (0.4%) which are again dominated by growing stevia, as opposed to transport. Finally, replacing ethanol from fermentation with that from fossil sources increased impact in some categories: GWP (1.4%), FWEu (1.2%), and FRS (37.9%). Fossil derived ethanol is responsible for a GWP of 3.23 kgCO_2_-eq/kg_RA60_ compared with fermentation at 2.75 kgCO_2_-eq/kg_RA60_, and FRS increase is due to fossil-derived ethylene extraction. Likewise, it is worth noting that land use does not reduce by much due to the dominance of cultivation on this impact category (accounting for 32.67 m^2^acrop-eq/kg_RA60_ out of a total 37.84 m^2^acrop-eq/kg_RA60_ in the base case), fermented ethanol only accounting for 4.41 m^2^acrop-eq/kg_RA60_.

## Sensitivity analysis

Table 1 shows sensitivity of the LCA impact assessment to uncertainty within life cycle inventory data. Uncertainty in background and foreground data was modelled as a Monte-Carlo simulation. The results are shown in terms of the originally calculated impact, mean of the uncertainty analysis, standard deviation, and relative standard deviation of the uncertainties. Relative standard deviation is defined as standard deviation as a percentage of the mean value. Two impact categories have notably high sensitivity: IR and WC. Regarding IR, over 50% of total impact derives from electricity consumed in France, the majority of which is from nuclear power (IEA 2021). An uncertainty analysis of the French electricity supply background process gave a similarly large relative standard deviation to that shown, indicating that much of the uncertainty derives from the background process. Variability in WC is due to large flows of water into and out of the system which partially cancel out one another, leading to lower net water consumption. Therefore, uncertainty in the individual flows results in a large relative variation compared with net flow.

**Table 1:** Sensitivity of results to uncertainties within process data, as a function of 1 kg_RA60_. Impact category abbreviations given in caption for Fig. 2

Impact category	Unit	Original	Mean	Standard deviation	Relative standard deviation
GWP	kgCO_2_-eq	20.25	20.23	1.70	8.39
SOD	kgCFC11-eq	2.06 × 10^−4^	2.06 × 10^−5^	5.94 × 10^−6^	2.89
IR	kBqCo-60-eq	0.77	0.77	1.07	139.6
OF,HH	kgNOx-eq	6.49 × 10^−2^	6.50 × 10^−2^	6.89 × 10^−3^	10.61
FPM	KgPM2.5-eq	3.92 × 10^−2^	3.93 × 10^−2^	2.93 × 10^−3^	7.47
OF,T	kgNOx-eq	6.88 × 10^−2^	6.88 × 10^−2^	6.94 × 10^−3^	10.01
TA	kgSO2-q	0.10	0.10	4.80 × 10^−3^	4.63
FWEu	kgP-eq	1.44 × 10^−2^	1.44 × 10^−2^	7.22 × 10^−3^	50.28
Meu	kgN-eq	2.47 × 10^−2^	2.47 × 10^−2^	7.58 × 10^−4^	3.07
TEc	kg1,4-DCB	1.44 × 10^−2^	1.44 × 10^−2^	32.63	22.68
FWEc	kg1,4-DCB	1.44	1.45	0.48	32.94
MEc	kg1,4-DCB	1.82	1.83	0.62	33.81
HCT	kg1,4-DCB	1.47	1.46	2.50	170.85
HnCT	kg1,4-DCB	34.83	34.79	12.55	36.07
LU	m2acrop-eq	37.85	37.81	1.65	4.36
MRS	kg-Cu-eq	9.95 × 10^−2^	9.94 × 10^−2^	9.92 × 10^−3^	9.98
FRS	kg-oil-eq	4.58	4.58	0.36	7.82
WC	m^3^	2.53	2.54	5.58	220.04

## Discussion

### Sweetness equivalence

Results presented in this study are for production of 1 kg of RA60. However, RA60 is never swapped with sugar on a like-for-like mass basis in any formulation. Instead, a smaller quantity of RA60 is used in place of a larger quantity of sugar. Therefore, an alternative way of understanding environmental impact of a sweetener is in terms of sucrose equivalence, or sweetness equivalence (both SE), i.e., mass of RA60 (0.004 g) required to produce the same sweetness as 1 kg of sugar (specifically sucrose). Sugar has an SE of 1.

**Fig. 6:**
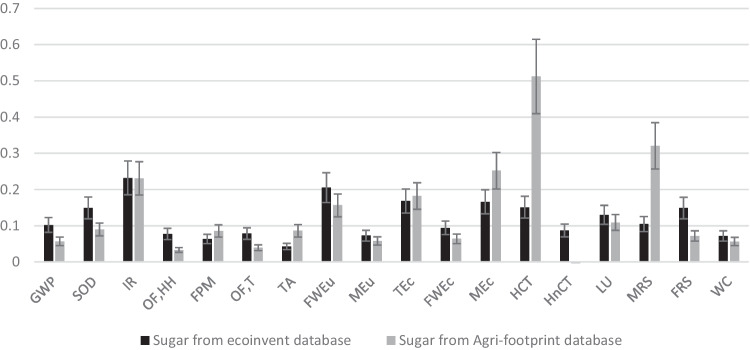
Relative impact of 1 kg_SE_ RA60 to a global mix of sugar (impact = 1 for each impact category), for sugar within the ecoinvent 3.8 database (black) and the Agri-footprint 6.1 database (grey). Effect of sweetness equivalence variation between 200 and 300 shown as error bars on RA60 data

Typically, SE values depend upon application of RA60 and sweetness that is required and tend to range between the low 200 s to the high 300 s (Cardello et al. 1999; Wallin 2004). Figure 6 shows the relative environmental impact of RA60 when 0.004 g is used to replace 1 kg sugar. Impact data for sugar is derived from two LCA databases, ecoinvent 3.8 (black bars), and Agri-footprint 6.1 (grey bars) for a global market mix of 80% sugar from cane and 20% sugar from beet (ISO 2020; OECD et al. 2021). The effect of an SE change from 200 to 300 (± 20%) for RA60 is also shown by the error bars. Supporting data are given in Supplementary Information, Table S4. The results show that production of RA60 has the potential to be less harmful to the environment than production of sugar for all levels of assumed SE in the ecoinvent database, and for all but the human non-carcinogenic toxicity (HnCT) impact category within the Agri-footprint 6.1 database. For example, for GWP, impact of RA60 is between 5.7% and 10.2% of the equivalent sweetness sugar, and for LU, it is 5.6% and 7.2%. For HnCT, the impact of sugar is negative, indicating that there is a net absorption of toxins such as heavy metals. However, it should be noted that, because this study is cradle-to-gate, HnCT might increase with inclusion of later life cycle processes, such as food manufacture, consumption, or waste disposal.

The closest study to which a comparison may be made for steviol glycoside production is Milovanoff and Kicak (2022). However, it should be noted that this study focuses on a steviol glycoside mixture with 60% Reb A content, whereas the target product in Milovanoff and Kicak (2022) is a more pure 97% Reb A. Therefore, comparison should be made with that in mind. If expressing results in terms of 1 kg_SE_, GWP of RA60 from this study is 8.1 × 10^−2^ kgCO_2_-eq/kg_SE_, compared with 8.3 × 10^−1^ kgCO2-eq/kg_SE_ as reported by Milovanoff and Kicak (2022). Similarly, for FWEu, impact calculated here is 5.8 × 10^−5^ kgP-eq/kg_SE_ compared with 1.57 × 10^−4^ kgP-eq/kg_SE_, and for LU, it is 1.5 × 10^−1^ m^2^acrop-eq/kg_SE_ compared with 2.5 × 10^−1^ m^2^acrop-eq/kg_SE_ respectively. Milovanoff and Kicak (2022) show a greater impact in each instance. However, this likely due to extra processing required to produce a more purified Reb A in that study and a reduced yield of final product from the stevia leaves.

### Functional use of steviol glycosides

It should be noted that comparisons presented in Section 6.1 are only valid for instances when sugar can be directly replaced with RA60 within a formulation. However, this is not always the case as replacement of added sugar might be accompanied by other formulation changes (e.g., inclusion of a bulking agent). The easiest swap is in a sweetened drink, where a large mass of sugar may be replaced with a small quantity of RA60. In contrast, for solid foods, removing a large quantity of sugar (and replacing it with a small quantity of RA60) can have a detrimental effect on the final product; technical function, including hydroscopic control or mouth-feel, of the sugar also needs to be replaced. Technical functions can be replicated using bulking agents, e.g., sorbitol or maltitol. However, the only bulking agent LCA studies that the authors are aware of are for sorbitol by Moreno et al. (2020) and Akmalina (2019). In terms of GWP these give impacts of 2.20 kgCO_2_-eq/kg to 5.09 kgCO_2_-eq/kg and 3.55 kgCO_2_-eq/kg respectively for production of sorbitol. The differing results from Moreno et al. (2020) arise from the methods used to produce the glucose precursor material (1.56 kgCO_2_-eq/kg for acid hydrolysis and 4.45 kgCO_2_-eq/kg for enzyme hydrolysis of maize starch into glucose).

To replace 1 kg of sugar in a food, approximately 4 g of RA60 is used alongside to 996 g of sorbitol. Using impact data from Moreno et al. (2020), net impact for the sorbitol/RA60 mix is 2.28 kgCO_2_-eq/kg and 5.15 kgCO_2_-eq/kg for low and high values of sorbitol production respectively. These numbers indicate that RA60 accounts for 3.5% to 1.6% net impact of creating bulk replacement for sugar based on sweetness and mass equivalence bases. Therefore, it is important to have a clearer understanding of potential impacts for bulking agents to make a full assessment of any formulation and any potential benefits of RA60.

### Transport

The production process within this study is for stevia leaves grown and dried in Greece and transported to France for extraction. The effect of moving extraction from France to Greece reduced the potential impact from 20.3 kgCO_2_-eq/kg_RA60_ to 16.4 kgCO_2_-eq/kg_RA60_, or 18.8%. The need for transport is historical, as the extraction factory existed in France when the collaboration with the Greek stevia cooperative began. However, the impact of international transport of dried leaves is recognized, and there are plans to mitigate this by performing extraction of crude SGs in Greece. This will reduce environmental impact of the RA60 production in future.

### Land use

Stevia in this study is produced by farmers who have transitioned from growing tobacco. Given that tobacco production is in decline around the world, particularly in Greece (FAOSTAT 2022), this indicates that upscaling of stevia production might not cause land use change, occupying instead land previously used for tobacco growing. Therefore, a further interesting comparison point for RA60 production is the amount of land used to supply 1 kg_SE_. Figure 6 shows that RA60 requires 0.15 m^2^acrop-eq/kg_SE_ compared with a global mix of sugar that requires 1.16 m^2^acrop-eq/kg_SE_ (ecoinvent) and 1.39 m^2^acrop-eq/kg_SE_ (Agri-footprint), a reduction of approximately 87% to 89%. This reflects the high SE of RA60, meaning that an annual mass yield of 360 kg_RA60_/ha equates to an SE yield of approximately 86 tSE/ha. For comparison, world average annual yield of sugar from beet is approximately 9.1 t_SE_/ha (based upon 16% sugar content (Duraisam et al. 2017) and 57.1 t/ha yield beet (Ritchie et al. 2021)). Likewise, sugar from cane might have an annual yield of 7.3 to 10.9 t_SE_/ha (based upon a 10% to 15% sugar content (Duraisam et al. 2017) and a yield of 72.6 t/ha (Ritchie et al. 2021)). These values indicate that SGs have potential to use less land footprint than sugar production for a given sweetness equivalence which shows promise from a land sparing perspective alone.

### Scale of production

Comparison of RA60 to sugar is complicated by the different scales of the two industries. Production scale of the case study business is approximately 7.2 t/yr_RA60_, and global production of SGs (not limited to RA60) is approximately 65 kt/yr (interview data). In contrast, global production of sugar is approximately 169.6 Mt/yr (ISO 2019), or four orders of magnitude larger. Commercial sugar production has a much longer history than that of SG production. Therefore, when considering environmental impact, it must be borne in mind that sugar production has been optimized over many years. Furthermore, RA60 production within this study is small scale, where farmers, working as part of a cooperative, cultivate on average 0.83 ha each. Upscaled cultivation of stevia might yet lead to efficiencies of scale and environmental impact from that presented in this study.

### Limitations and further research

This study explored environmental impact of producing purified RA60 from stevia leaves cultivated in Greece. However, there are some limitations to the study, some of which could be explored in future research:

- The present study focused on an indicative stevia leaf that has a 10% SG content by dry matter mass (after extraction and purification) and a yield of 3.6 t/ha dry leaves. However, there are different varieties of stevia which have been developed (Angelini et al. 2018) to maximize production of a particular rebaudioside or optimize yield for a given local climate. Each variety has different yields in terms of mass of leaves per hectare or SGs per kg of leaf. Therefore, in future, studies could explore impact of variability in these factors in determining environmental impact of different SG mixes.
- This study showed the impact of producing RA60 from cradle-to-gate; it does not include incorporation of the ingredient into food or beverage products, nor consumption and waste disposal. Such a cradle-to-grave life cycle was outside of the scope of this study. A full life cycle study will also need to incorporate health implications for replacing added sugar with sweeteners within diets. This is another area for future study.

## Conclusions

The environmental life cycle assessment for producing a mixture of SGs with a rebaudioside A content of 60% and purity of 95% (RA60) from leaf is presented in this study. RA60 is used in food and beverage products to replace the sweetness of added sugar. Results showed that in terms of environmental impact of replacing sweetness of sugar, RA60 compared favorably with sugar. The study presented cradle-to-gate results for producing RA60, but final use of RA60 is an important further consideration. A “simple” swap of RA60 for sugar may be achieved in beverages, which accounts for the majority of global sweetener use at present. However, with increased interest in the use of sweeteners in solid formulations to combat non-communicative diseases, there is a need to better understand how sugar might be replaced within solid formulations. In such instances, RA60 needs to be combined with bulking agents that can replace the technical functions of added sugar. The environmental impact of bulking agents is not well studied at present. In parallel, it is also necessary to understand the potential health benefits for consumers in replacing added sugar with sweeteners and to understand any subsequent changes in environmental impact of a positive health change. Therefore, this research highlights that while RA60 reduces environmental impact when replacing the sweetness of sugar, further research is needed on the full life cycle of the products and diets in which RA60 is consumed.

## Supplementary Information

Below is the link to the electronic supplementary material.

Supplementary file1 (DOCX 51 KB)

## Data Availability

All data generated or analyzed during this study are included in this published article and its supplementary information files.

## Declarations

### Disclaimer

The material presented and views expressed here are the responsibility of the author(s) only. The EU Commission takes no responsibility for any use made of the information set out.
