# Supplementary material for: Environmental life cycle assessment of production of the high intensity sweetener steviol glycosides from Stevia rebaudiana leaf grown in Europe: The SWEET project
Source: Int J Life Cycle Assess. 2023 Jan 14;28(3):221–33. doi: 10.1007/s11367-022-02127-9 (PMC9839952; doi:10.1007/s11367-022-02127-9)
Supplement: Supplementary file 1 — Supplementary file1 (DOCX 51 KB) [file 11367_2022_2127_MOESM1_ESM.docx]

# Supplementary information: Environmental life cycle assessment of production of the high intensity sweetener steviol glycosides from Stevia rebaudiana leaf grown in Europe: The SWEET project

## Authors

J. Suckling^1^*, S. Morse^1^, R. Murphy^1^, S. Astley^2^, J.C.G. Halford^3,4^, J.A. Harrold^4^, A. Le-Bail^5^, E. Koukouna^6^, H. Musinovic^2^, J. Perret^7^, A. Raben^8^, M. Roe^2^, J. Scholten^6^, C. Scott^9^, C. Stamatis^10^, C. Westbroek^6^

^1^Centre for Environment and Sustainability, University of Surrey, Guildford, UK

^2^EuroFIR AISBL, Brussels, Belgium

^3^School of Psychology, University of Leeds, Leeds, UK

^4^Department of Psychology, University of Liverpool, Liverpool, UK

^5^ONIRIS, UMR GEPEA CNRS 6144, Nantes, France

^6^Blonk Consultants, Gouda, The Netherlands

^7^Stevia Natura, Auvergne, France

^8^Department of Nutrition, Exercise and Sports, University of Copenhagen, Copenhagen, and Clinical Research, Copenhagen University Hospital – Steno Diabetes Center Copenhagen, Herlev, Denmark

^9^Cargill, Plymouth, Minnesota, USA

^10^Stevia Hellas, Lamia, Greece

*corresponding author

## Acknowledgements

The project has received funding from the European Union’s Horizon 2020 research and innovation programme under grant agreement No 774293. The material presented and views expressed here are the responsibility of the author(s) only. The EU Commission takes no responsibility for any use made of the information set out.

Table S1 Numerical data for each impact category as a function of life cycle phase. Data supports Figure 2 in the manuscript.

| **Impact category** | **Unit** | **Total** | **Seedlings** | **Field Production** | **Processing** | **Extraction** |
| --- | --- | --- | --- | --- | --- | --- |
| GWP | kg CO2 eq | 2.02E+01 | 1.45E-01 | 1.04E+01 | 1.26E+00 | 8.49E+00 |
| SOD | kg CFC11 eq | 2.06E-04 | 4.66E-08 | 1.29E-04 | 7.30E-07 | 7.61E-05 |
| IR | kBq Co-60 eq | 7.66E-01 | 9.60E-03 | 2.27E-01 | 5.79E-02 | 4.72E-01 |
| OF,HH | kg NOx eq | 6.49E-02 | 2.62E-04 | 2.09E-02 | 2.66E-03 | 4.11E-02 |
| FPM | kg PM2.5 eq | 3.92E-02 | 1.62E-04 | 1.91E-02 | 2.30E-03 | 1.76E-02 |
| OF,T | kg NOx eq | 6.88E-02 | 2.75E-04 | 2.15E-02 | 2.72E-03 | 4.43E-02 |
| TA | kg SO2 eq | 1.04E-01 | 4.48E-04 | 4.23E-02 | 5.05E-03 | 5.58E-02 |
| FWEu | kg P eq | 1.44E-02 | 6.20E-05 | 1.03E-02 | 1.92E-03 | 2.13E-03 |
| MEu | kg N eq | 2.47E-02 | 5.14E-06 | 5.73E-03 | 2.80E-04 | 1.87E-02 |
| TEc | kg 1,4-DCB | 1.44E+02 | 5.13E-01 | 7.30E+01 | 4.14E+00 | 6.59E+01 |
| FWEc | kg 1,4-DCB | 1.44E+00 | 1.09E-02 | 1.06E+00 | 9.84E-02 | 2.74E-01 |
| MEc | kg 1,4-DCB | 1.82E+00 | 1.52E-02 | 1.38E+00 | 1.30E-01 | 2.98E-01 |
| HCT | kg 1,4-DCB | 1.47E+00 | 2.51E-02 | 9.96E-01 | 1.17E-01 | 3.30E-01 |
| HnCT | kg 1,4-DCB | 3.48E+01 | 2.71E-01 | 2.01E+01 | 2.41E+00 | 1.21E+01 |
| LU | m2a crop eq | 3.78E+01 | 2.99E-03 | 3.35E+01 | 1.68E-01 | 4.19E+00 |
| MRS | kg Cu eq | 9.95E-02 | 1.05E-03 | 7.25E-02 | 2.32E-03 | 2.36E-02 |
| FRS | kg oil eq | 4.58E+00 | 4.86E-02 | 2.10E+00 | 3.89E-01 | 2.04E+00 |
| WC | m3 | 2.53E+00 | 2.09E-03 | 2.11E+00 | 9.63E-03 | 4.14E-01 |

Table S2 Numerical data for each impact category as a function of life cycle phase, with impact normalised to the phase with greatest contribution. Data supports Figure 3 in the manuscript.

| **Impact category** | **Unit** | **Seedlings** | **Field Production** | **Processing** | **Extraction** |
| --- | --- | --- | --- | --- | --- |
| GWP | kg CO2 eq | 1.40E-02 | 1 | 1.22E-01 | 8.20E-01 |
| SOD | kg CFC11 eq | 3.62E-04 | 1 | 5.67E-03 | 5.91E-01 |
| IR | kBq Co-60 eq | 2.03E-02 | 4.81E-01 | 1.23E-01 | 1 |
| OF,HH | kg NOx eq | 6.36E-03 | 5.08E-01 | 6.46E-02 | 1 |
| FPM | kg PM2.5 eq | 8.50E-03 | 1 | 1.20E-01 | 9.23E-01 |
| OF,T | kg NOx eq | 6.22E-03 | 4.86E-01 | 6.13E-02 | 1 |
| TA | kg SO2 eq | 8.04E-03 | 7.58E-01 | 9.05E-02 | 1 |
| FWEu | kg P eq | 6.02E-03 | 1 | 1.86E-01 | 2.07E-01 |
| MEu | kg N eq | 2.75E-04 | 3.06E-01 | 1.50E-02 | 1 |
| TEc | kg 1,4-DCB | 7.02E-03 | 1 | 5.67E-02 | 9.02E-01 |
| FWEc | kg 1,4-DCB | 1.03E-02 | 1 | 9.28E-02 | 2.58E-01 |
| MEc | kg 1,4-DCB | 1.10E-02 | 1 | 9.39E-02 | 2.16E-01 |
| HCT | kg 1,4-DCB | 2.52E-02 | 1 | 1.17E-01 | 3.31E-01 |
| HnCT | kg 1,4-DCB | 1.35E-02 | 1 | 1.20E-01 | 6.00E-01 |
| LU | m2a crop eq | 8.93E-05 | 1 | 5.02E-03 | 1.25E-01 |
| MRS | kg Cu eq | 1.45E-02 | 1 | 3.20E-02 | 3.25E-01 |
| FRS | kg oil eq | 2.31E-02 | 1 | 1.85E-01 | 9.71E-01 |
| WC | m3 | 9.92E-04 | 1 | 4.57E-03 | 1.96E-01 |

Table S3 Numerical data for each impact category for each of the sensitivity scenarios. Data supports Figure 5 in the manuscript.

| **Impact category** | **Unit** | **Original** | **Air Drying** | **No Transport** | **Fossil Ethanol** |
| --- | --- | --- | --- | --- | --- |
| GWP | kg CO2 eq | 2.02E+01 | 1.75E+01 | 1.64E+01 | 2.05E+01 |
| SOD | kg CFC11 eq | 2.06E-04 | 2.05E-04 | 2.03E-04 | 1.82E-04 |
| IR | kBq Co-60 eq | 7.66E-01 | 7.00E-01 | 3.81E-01 | 7.32E-01 |
| OF,HH | kg NOx eq | 6.49E-02 | 6.15E-02 | 3.89E-02 | 6.09E-02 |
| FPM | kg PM2.5 eq | 3.92E-02 | 3.31E-02 | 3.05E-02 | 3.41E-02 |
| OF,T | kg NOx eq | 6.88E-02 | 6.53E-02 | 4.24E-02 | 6.53E-02 |
| TA | kg SO2 eq | 1.04E-01 | 9.09E-02 | 7.84E-02 | 8.35E-02 |
| FWEu | kg P eq | 1.44E-02 | 8.37E-03 | 1.43E-02 | 1.46E-02 |
| MEu | kg N eq | 2.47E-02 | 2.44E-02 | 2.46E-02 | 1.95E-02 |
| TEc | kg 1,4-DCB | 1.44E+02 | 1.36E+02 | 8.97E+01 | 1.42E+02 |
| FWEc | kg 1,4-DCB | 1.44E+00 | 1.17E+00 | 1.39E+00 | 1.38E+00 |
| MEc | kg 1,4-DCB | 1.82E+00 | 1.46E+00 | 1.72E+00 | 1.80E+00 |
| HCT | kg 1,4-DCB | 1.47E+00 | 1.14E+00 | 1.34E+00 | 1.45E+00 |
| HnCT | kg 1,4-DCB | 3.48E+01 | 2.81E+01 | 3.32E+01 | 3.21E+01 |
| LU | m2a crop eq | 3.78E+01 | 3.78E+01 | 3.75E+01 | 3.42E+01 |
| MRS | kg Cu eq | 9.95E-02 | 9.68E-02 | 9.09E-02 | 9.38E-02 |
| FRS | kg oil eq | 4.58E+00 | 3.69E+00 | 3.34E+00 | 6.32E+00 |
| WC | m3 | 2.53E+00 | 2.52E+00 | 2.52E+00 | 2.18E+00 |

Table S4 Numerical data for each impact category for each of sugar from various sources and 1 kg sucrose equivalence of RA60. Sugar data as per the ecoinvent 3.8 and Agri-footprint 6.1 database. Data supports Figure 6 in the manuscript.

| **Impact category** | **Unit** | **Sugar from Agri-footprint 6.1 (1 kg_SE_)** | **Sugar from ecoinvent 3.8 (1kg_SE_)** | **RA60 (1 kg_SE_)** |
| --- | --- | --- | --- | --- |
| GWP | kg CO2 eq | 1.43E+00 | 7.93E-01 | 8.10E-02 |
| SOD | kg CFC11 eq | 9.18E-06 | 5.51E-06 | 8.23E-07 |
| IR | kBq Co-60 eq | 1.33E-02 | 1.32E-02 | 3.06E-03 |
| OF,HH | kg NOx eq | 7.88E-03 | 3.35E-03 | 2.60E-04 |
| FPM | kg PM2.5 eq | 1.83E-03 | 2.46E-03 | 1.57E-04 |
| OF,T | kg NOx eq | 7.01E-03 | 3.51E-03 | 2.75E-04 |
| TA | kg SO2 eq | 4.79E-03 | 9.61E-03 | 4.14E-04 |
| FWEu | kg P eq | 3.68E-04 | 2.81E-04 | 5.76E-05 |
| MEu | kg N eq | 1.71E-03 | 1.36E-03 | 9.89E-05 |
| TEc | kg 1,4-DCB | 3.15E+00 | 3.41E+00 | 5.74E-01 |
| FWEc | kg 1,4-DCB | 9.01E-02 | 6.14E-02 | 5.77E-03 |
| MEc | kg 1,4-DCB | 2.89E-02 | 4.39E-02 | 7.29E-03 |
| HCT | kg 1,4-DCB | 1.15E-02 | 3.88E-02 | 5.87E-03 |
| HnCT | kg 1,4-DCB | -1.26E-01 | 1.60E+00 | 1.39E-01 |
| LU | m2a crop eq | 1.39E+00 | 1.16E+00 | 1.51E-01 |
| MRS | kg Cu eq | 1.24E-03 | 3.80E-03 | 3.98E-04 |
| FRS | kg oil eq | 2.55E-01 | 1.23E-01 | 1.83E-02 |
| WC | m3 | 1.79E-01 | 1.41E-01 | 1.01E-02 |

Table S5 Emissions data from fertilizer use and crop residues. Data derived from inventory data presented in Sections 3.2 (Phase: Field cultivation), 3.3 (Phase: Leaf processing), and 3.4 (Phase: Extraction) in the manuscript.

| **Domain** | **Source** | **Emission** | **Greece (kg/18t leaves)** | **Greece (mg/18t leaves)** | **France (kg/kgRA60)** | **France (mg/kgRA60)** |
| --- | --- | --- | --- | --- | --- | --- |
| Air | Fertiliser | N2O direct | 3.03 |  |  |  |
|  |  | N2O via NO | 7.71 |  |  |  |
|  |  | N2O via NH3 & NOx | 0.33 |  |  |  |
|  |  | N2O via NO3 leach | 0.80 |  |  |  |
|  |  | NH3 direct | 25.76 |  |  |  |
|  |  | CO2 direct | 58.92 |  |  |  |
|  | Crop residue | N2O direct | 4.31 |  | 0.0034 |  |
|  |  | N2O via NO3 leach | 1.14 |  | 0.00090 |  |
| Water | Fertiliser | NO3 direct | 48.78 |  |  |  |
|  | Crop residues | NO3 direct | 69.28 |  | 0.18 |  |
| Groundwater | Fertiliser & crop residues | Cd |  | 250 |  |  |
|  |  | Cu |  | 18000 |  |  |
|  |  | Zn |  | 165000 |  |  |
|  |  | Pb |  | 3000 |  |  |
|  |  | Ni |  | 0 |  |  |
|  |  | Cr |  | 106000 |  |  |
|  |  | Hg |  | 6.5 |  |  |
| Soil | Fertiliser | P direct | 9.32 |  |  |  |
|  | Fertiliser & crop residues | Cd |  | 7499 |  |  |
|  |  | Cu |  | -28296 |  |  |
|  |  | Zn |  | -167574 |  |  |
|  |  | Pb |  | 18627 |  |  |
|  |  | Ni |  | 16281 |  |  |
|  |  | Cr |  | 686 |  |  |
|  |  | Hg |  | 19.6 |  |  |
|  | Crop residues | Cd |  |  |  | 0.3 |
|  |  | Cu |  |  |  | 21 |
|  |  | Zn |  |  |  | 125 |
|  |  | Pb |  |  |  | 0.3 |
|  |  | Ni |  |  |  | 2.5 |
|  |  | Cr |  |  |  | 4 |
|  |  | Hg |  |  |  | 0.03 |

Table S6 Inventory data for seedling propagation in greenhouses. Data per 247 seedlings.

| **Substance name** | **Quantity** | **Unit** |
| --- | --- | --- |
| **Outputs to technosphere** | | |
| Stevia seedlings | 247 | p |
| **Inputs from technosphere** | | |
| Compost | 2 | kg |
| Polystyrene foam slab | 0.33 | kg |
| Polystyrene scrap, post-consumer | 0.33 | kg |
| Tap water | 2.5 | kg |
| Mower use for seedling topping | 0.000048 | ha |
| Greenhouse | 0.3 | m2 |
| Plastic liner | 0.0624 | kg |
| Extrusion of plastic liner | 0.0624 | kg |
| Fertiliser | 0.0018 | kg |
| Agricultural machinery | 1.64E10-6 | kg |
| **Inputs from energy** | | |
| Electricity (GR) | 0.0171 | kWh |
| **Waste processes** | | |
| Waste plastic liner | 0.0624 | kg |

Table S7 Inventory data for field cultivation and leaf drying. Data per 18 tonnes dried leaves.

| **Substance name** | **Quantity** | **Unit** |
| --- | --- | --- |
| **Outputs to technosphere** | | |
| Stevia leaves, dried | 18 | t |
| Stevia plant stems | 27 | t |
| **Inputs from nature** | | |
| Land change | 10000 | m2 |
| Land use | 50000 | m2a |
| **Inputs from technosphere** | | |
| Stevia seedling | 65000 | p |
| Irrigation | 3150 | m3 |
| Tobacco planter | 16 | kg |
| Combine harvester | 80 | kg |
| Weeding machine | 45 | kg |
| Copper sulphate spreader | 16 | kg |
| Tractor | 67.28 | kg |
| Diesel | 4076.0741 | kWh |
| Copper sulphate | 42.19 | kg |
| Fertiliser | 1200 | kg |
| Leaf drying shed | 2.97 | m2 |
| **Inputs from energy** | | |
| Electricity (GR) | 5400 | kWh |
| **Waste processes** | | |
| Scrap steel | 224.28 | kg |

Table S8 Inventory data for leaf processing. Data given per 150 kg dried leaves.

| **Substance name** | **Quantity** | **Unit** | **Further information** |
| --- | --- | --- | --- |
| **Outputs to technosphere** | | | |
| Stevia leaves, processed for transport to RA60 extraction factory | 51 | kg | 40% allocation. |
| Stevia leaves, processed for tea | 76.5 | kg | 60% allocation. |
| Stevia leaf powder | 18 | kg | 0% allocation. |
| Stems and stones | 4.5 | kg | 0% allocation. |
| **Inputs from technosphere** | | | |
| Dried stevia leaves, from fields | 150 | kg |  |
| Zig-zag cleaner | 0.002 | kg |  |
| Cleaning facility | 6.94E-08 | m2 |  |
| Cardboard box | 5.06 | kg |  |
| Transport by tractor and trailer | 0.75 | tkm |  |
| Transport by light commercial vehicle | 0.75 | tkm |  |
| **Inputs from energy** | | | |
| Electricity (GR) | 12 | kWh |  |

Table S9 Inventory data for RA60 extraction. Data given per 1 kg RA60.

| **Substance name** | **Quantity** | **Unit** | **Further information** |
| --- | --- | --- | --- |
| **Outputs to technosphere** | | | |
| R60 | 1 | kg | 100% allocation. |
| Plant residues | 9 | kg | 0% allocation. |
| **Inputs from technosphere** | | | |
| Stevia leaves | 10 | kg |  |
| Water (de-ionised) | 100 | kg |  |
| Lime | 0.02 | kg |  |
| Citric acid | 0.02 | kg |  |
| Ethanol, from fermentation, for extraction | 2.367 | kg |  |
| Ethanol, for purification | 0.03 | kg |  |
| Ethanol, re-purification | 3.5 | kg |  |
| Sodium silicate | 0.132 | kg |  |
| Factory | 3.38E-11 | p |  |
| Extrusion of plastic film | 1 | g |  |
| Corrugated carboard box | 39.65 | g |  |
| Lorry freight transport | 17 | tkm |  |
| Sea freight transport | 9 | tkm |  |
| Tractor and trailer transport | 0.09 | tkm |  |
| **Inputs from energy** | | | |
| Heat | 0.5 | kWh |  |
| Electricity (FR) | 0.5 | kWh |  |
| **Emissions to air** | | | |
| Ethanol | 0.03 | kg |  |
| **Emissions to water** | | | |
| Ethanol | 3 | kg |  |
| **Waste processes** | | | |
| Wastewater | 0.1 | m3 |  |
| Spent resin | 0.0132 | kg |  |
| Cardboard waste | 396.5 | g |  |
